# Grain Self-Sufficiency in Guangdong, China: Current Trends and Future Perspectives

**DOI:** 10.3390/foods14071126

**Published:** 2025-03-25

**Authors:** Yi Xiao, Jianya Zhao, Yanglan Zhang, Shu Wang

**Affiliations:** 1 Department of Science and Technology Research, Jinan University, 855 Xingye E. Ave., Guangzhou 510632, China; 2 Jinan University-University of Birmingham Joint Institute, Jinan University, 855 Xingye E. Ave., Guangzhou 511436, China; 3 School of Economics, Jinan University, 601 W. Huangpu Ave., Guangzhou 510632, China

**Keywords:** grain self-sufficiency, food security, China

## Abstract

The rising number of disturbances caused by natural hazards, epidemics, and international conflicts seriously threatens global agriculture and food systems. In order to combat the increasing uncertainty, the Chinese government proposed a “dual circulation” strategy, which puts forward a new requirement of promoting self-sufficiency. Among various food categories, grain serves as a cornerstone for sustaining human life during public crises. Guangdong Province is one of the most economically developed and highly densely populated regions in China; therefore, it faces significant pressure to enhance food security. This study investigates the spatial and temporal patterns of the rate of grain self-sufficiency (RSSG) at the county levels in Guangdong Province from 2014 to 2023. The findings reveal the following: (1) rice remains the predominant grain crop, with its sown area and production consistently accounting for over 75%; (2) the RSSG is 28.14% on average, which is lower than the governmental goal of 30%; (3) the RSSG has significant regional disparities and distinct agglomeration patterns; and (4) scenario simulations indicate that grain yield improvement and grain loss reduction have the potential to promote the RSSG to 34.14%. Based on the research findings, this paper proposes the following policy recommendations: (1) prioritize farmland preservation and implement the “storing grain in the land” strategy to ensure food supply; (2) adopt advanced agricultural technologies under the “storing grain in the technology” strategy to improve grain yield; (3) reduce grain loss by strengthening disaster prevention, improving storage management, and enhancing storage efficiency; and (4) strengthen interdepartmental coordination. The integrated application of these strategies will help Guangdong Province enhance its grain self-sufficiency, ensure food security, and contribute to the achievement of national food security goals.

## 1. Introduction

Since 2020, the rising number of internal and external shocks from natural hazards, epidemics, and international conflicts demonstrate the frailty and vulnerability of the global agriculture and food system. Extreme weather events such as drought and heavy rain, favored by climate change, have a negative impact on crop yields [1,2]. Since 2020, the world has grappled with the coronavirus 2019 (COVID-19) pandemic and the Russia–Ukraine war, both of which have had significantly adverse influences on food accessibility [3]. From early 2020, with the widespread transmission of COVID-19, extensive disruptions to food systems were noted [4]. Agricultural production had been disrupted by the shortage of input, such as labor and agricultural materials [5]. Ultimately, the vulnerable food supply chain during the shutdown period resulted in household food insecurity [6,7,8]. Just as pressures from the COVID-19 crisis began to wane, the Russia–Ukraine conflict disrupted grain exports from Russia’s Black Sea region and caused a considerable rise in food prices [9,10]. Apart from the direct military conflict as the most significant actor in the global agriculture system [11], the China–U.S. trade disputes have also caused severe damage to agriculture and the food supply [12]. Such conflicts have been suggested to become more tense because Donald Trump’s government in the U.S. has adopted an approach of weaponizing food supplies [13]. Therefore, these factors exacerbate the vulnerability of agriculture and the food system and put a heavy threat on global food security.

In order to combat rising uncertainties, the Chinese government proposed the spirit of “holding the rice bowl of the Chinese people in their own hands firmly”, which emphasizes the importance of maintaining food self-sufficiency as the central theme of China’s food security [14]. As one of the largest agricultural countries globally, China grapples with multifaceted challenges in agricultural production. These challenges include cropland scarcity, water scarcity, non-point source pollution from chemical fertilizers, and the looming impacts of climate change. The challenges outlined pose significant barriers to the sustainable development of agriculture, potentially endangering national food security and the welfare of farmers in the long term. Therefore, China’s central government issued the No.1 document in 2014, declaring a break away from self-sufficiency to a new food security strategy—one that relies on domestic production with moderate imports [15]. China was a net exporter of maize until the 1990s, but since 2009, it has become a constant importer, with the import volume reaching 28 million tons in 2021 [15]. Consequently, China has become the worlds’ largest importer of maize [15]. In 2022, China’s total crop imports amounted to 164 million tons, representing an effective import of 0.8 billion hectares of arable land [16].

However, the “import substitution” policy should establish the stable international environment and free trade. Because of the increasing complexity of the international environment and the globalization of environmental governance, China’s agricultural trade is facing an increasing number of difficulties and challenges [17]. Crop failure exacerbated by extreme weather [18], food processing, and logistics suspended by public health emergencies [4], trade conflicts [12], and even worse, the war [9,10], is threatening stable and smooth food import. China has foreseen that “The world is undergoing profound changes unseen in a century”. In May 2020, China proposed the domestic and international “dual circulation” strategy, which refers to the domestic cycle as the mainstay, while domestic and international dual circulation can mutually promote and benefit each other [19,20]. With such a movement, the urgency and importance of self-sufficiency is reinforced again. For the purposes of sustaining human life, food variety has to be limited [21], and grain supply should have high priority [22,23,24].

**Figure 1:**
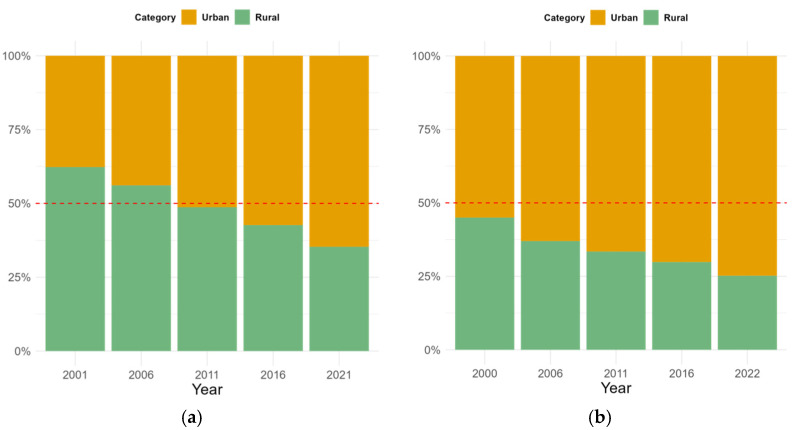
Ratios of urbanization: (**a**) China; (**b**) Guangdong province. Data Source: China Statistical Yearbook [40].

As the one of the largest developing countries in the world, China also suffers from strict regulations for ensuring national food security [24,25,26,27,28]. China’s vast territory is characterized by significant regional disparities [29,30,31,32], with Guangdong Province being particularly representative. Guangdong is one of the most economically developed regions in China, with rapid urbanization and industrialization [24,25]. Since the reformation and the opening up of the province, urbanization and economic growth in Guangdong have been rapidly promoted (see Figure 1). However, rapid urbanization and industrialization have severe negative impacts on food security and environmental sustainability. Industrialization is attracting large numbers of migrants to Guangdong, and the demand for food surges dramatically, while the agricultural endowments (farmland, water, etc.) are limited. The increase in agriculture cannot keep pace with the population growth [33,34,35]. Urbanization leads to significant land use changes, including the destruction of farmland, which affects its carrying capacity and food security [36]. Meanwhile, Guangdong faces serious internal regional economic disparities [37]. Guangdong is divided into four parts: east (Shantou, Shanwei, Chao Yang, Jieyang), west (Yangjiang, Zhanjiang, Maoming), north (Shaoguan, Heyuan, Meizhou, Qingyuan, Yunfu) and Pearl River Delta (Guangzhou, Zhuhai, Foshan, Huizhou, Jiangmen, Zhaoqing, Dongguan, Zhongshan). Located in the heart position of Guangdong, the Guangdong–Hong Kong–Macau Big Bay Area (Pearl River Delta plus Hong Kong SAR and Macau SAR) is a major economic hub in China. It is famous around the world, with two major international cities: Hong Kong and Macau [38]. On the other hand, the varied topographies, ranging from mountainous areas to the Pearl River Delta [39], involve certain endowments that result in different economic development and agricultural practices. Although the remaining parts of Guangdong have great gaps with the Big Bay Area, the regions’ reliance on the other parts for resources, such as water and food, makes great contributions to the achievement of the Big Bay Area for the remarkable and impressive economic development. In addition, as one of the pioneers of reform and opening up, Guangdong has built up well-established statistical systems, and the available data are comparatively comprehensive.

Previous studies have independently considered several problems and solutions of food security from different perspectives. Yang et al. (2024) [39] presents an “element coupling–function synergy” analytical framework to assess the multifunctionality of cultivated land resources in Guangdong, China, revealing trade-offs and synergies among various functions such as food production, ecological regulation, and social security, and proposing multi-objective management plans for sustainable land use. Mutelo et al. (2023) [41] employs high-resolution remote sensing to monitor the destruction of high-quality farmland in Guangdong, revealing significant conversion of farmland to industrial and residential uses, which poses a threat to food security due to the loss of fertile land. Fang et al. (2023) [36] evaluates the carrying capacity of cultivated land in Guangdong, using the entropy weight method; they found that ecological and environmental pressures have a significant impact on capacity, while economic and social factors remain stable. This observation highlights the need for green recovery strategies to improve land productivity and support sustainable agriculture. Chen et al. (2022) [42] quantifies the net carbon sequestration value of the rice production system in Guangdong, finding that compensating for this value could alleviate farmland abandonment and enhance food security and carbon neutrality. The study also points out that the self-sufficiency rate of rice in Guangdong was only about 22% in 2019, highlighting a significant food security crisis.

However, the potential risks of food shortages arising from public crises have yet to be fully explored and should not be overlooked. In 2019, Guangdong Province released a plan for the rural revitalization strategy, and a the goal of ensuring that grain self-sufficiency exceeds 30% [43]. Therefore, in this study, we collected data on grain production and consumption in Guangdong, assessing the self-sufficiency of grain; accordingly, we propose a practical pathway for further improvement. In this study, we aim to investigate the spatial and temporal patterns of the rate of grain self-sufficiency (RSSG) at the county level in Guangdong Province from 2014 to 2022; additionally, we analyze how grain yield improvement and grain loss reduction can promote the self-sufficiency of grain in Guangdong. Addressing Guangdong’s food security issues and ensuring the goal of keeping “the Chinese rice bowl firmly in their own hands” are important contributions to achieving the stable and sustainable development of countries and regions around the world.

## 2. Data and Methods

### 2.1. Method

Grain self-sufficiency can be assessed using various metrics, such as caloric value, production volume, or monetary value [44,45]. To evaluate the balance between grain production and consumption, the most intuitive and straightforward approach is to compare actual grain production with the region’s standard per capita grain consumption and the population it supports. By calculating either the gap (difference) or the ratio (division) between production and consumption, the local grain supply can be effectively assessed, and potential risks to grain self-sufficiency can be identified. This method [45,46] provides a practical framework for analyzing regional food security dynamics and identifying areas where localized interventions may be needed to address imbalances.

This study analyzes the grain self-sufficiency at the county/district level in Guangdong Province. For the assessment of regional food security, a micro-scale approach (at the county level) is more accurate than a macro-scale approach (at the provincial or municipal levels) [47]. County-level units play a key role in agricultural production and food security management. County governments are responsible for implementing policies and directly influencing grain planting and yield management [48]. Given China’s vast territory and significant regional disparities, studying county-level units helps to identify unique challenges and potentials in different areas, contributing to the improvement of grain production capacity across regions and thus promoting overall food security. The role of county-level units is particularly critical in responding to disasters and climate change. Therefore, studying grain self-sufficiency at the county level provides important support for the national food security strategy [49]. The analysis is based on the fundamental concepts of division and subtraction between grain production and consumption:

(1) $${RSSG}_{i,y}=\frac{Y_{i,y}}{S\times P_{i,y}}$$

(2) $${GSSG}_{i,y}=Y_{i,y}-(S\times P_{i,y})$$

where *RSSG_i,y_* (unit: %) indicates the ratio of self-sufficiency of grain in region, *i*, of year, *y*; *Y_i,y_* (unit: kg/ha) is grain production in county, *i*, of year, *y*; *S* (unit: kg grain/person*year) is the standard of individual grain consumption per year; *P_i,y_* (unit: person) is the population in region, *i*, in year, *y*. *GSSG_i,y_* (unit: %) indicates the gap of self-sufficiency of grain in region, *i*, of year, *y*. If *GSSG_i,y_* is positive, it indicates that the grain production in the region exceeds the local consumption needs, ensuring self-sufficiency. Conversely, a negative *GSSG_i,y_* implies a grain deficit, requiring imports from other regions (via domestic allocation or international trade). Several factors influence the calculation of actual grain production and consumption, including grain loss during seeding, harvesting, and storage. Apart from direct consumption for food, grain is also consumed indirectly for forage and food processing. These factors act as additional constraints and are often estimated using conversion coefficients. For example, grain loss accounts for approximately 12% of total production [50,51]. The security line for grain has been set to 400 kg per capita annually [52]. This methodological framework provides a structured approach to quantifying grain self-sufficiency and identifying regional disparities in grain supply, which are critical for ensuring food security.

### 2.2. Statistical Analytical Framework

**Figure 2:**
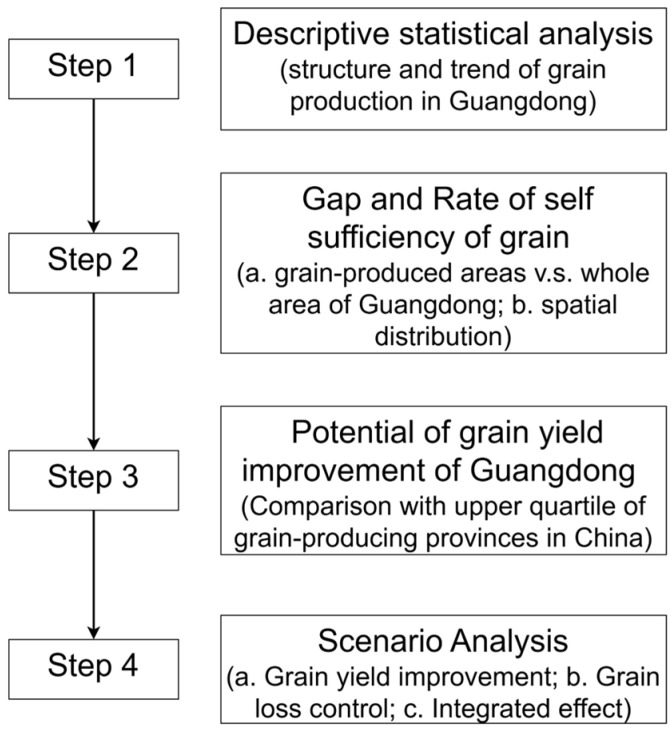
Data analysis procedure in this study.

The analyses in this study were conducted in four distinct stages (Figure 2). First, a descriptive statistical analysis was performed to identify the structure and trends in grain production in Guangdong.

Second, the study examined the gap and rate of grain self-sufficiency (*GSSG*, *RSSG*) in both grain-producing areas and the entire province of Guangdong Province, based on current yields. This analysis provides valuable insights into the current state of grain self-sufficiency in Guangdong. To mitigate potential bias from extreme one-year values, the average yield data from 2014 to 2023 were used. Additionally, the spatial distribution of grain self-sufficiency was analyzed to reveal the patterns of grain production and consumption across Guangdong.

Third, the potential for improving grain yields in Guangdong was assessed. As one of China’s leading economic provinces, Guangdong has significant potential to increase grain yield through a “storing grain to technology” strategy, which provides practical solutions. It is projected that Guangdong’s grain yield could reach the upper quartile level among grain-producing provinces in China. The equation for calculating the potential improvement in grain yield in Guangdong is as follows:

(3) $$Improvement=\frac{Y_{Q3,y}-Y_{GD,y}}{Y_{GD,y}}$$

where *Improvement* (unit: %) represents the potential grain yield improvement in Guangdong; *Y_Q3,y_* (unit: %) denotes the grain yield in the upper quartile of China’s grain-producing provinces for year, *y*; and *Y_GD,y_* (unit: %) is the grain yield in Guangdong for year, *y*.

Finally, based on the potential for grain yield improvement and the control of grain loss, this study employed a scenario analysis to explore pathways for strengthening grain security in Guangdong. The equation to calculate the change in grain production in Guangdong is as follows:

(4) $$Y_{i,y}^{*}=Y_{i,y}\times (1+Improvement)\times (1-Loss)$$

where *Loss* (unit: %) represents the grain loss, using a general figure for China (12%). In the grain loss control and grain yield improvement scenario, area-related parameters are adopted and discussed in Section 3.4. In the integrated scenario, both parameters were combined to generate new results.

### 2.3. Data

The primary data source for analyzing grain self-sufficiency in China is the *China Statistical Yearbook* [40,46,53,54,55,56,57,58]. In addition, the *Guangdong Statistical Yearbook* [59] serves as supplementary material [15]. Benefiting from a comprehensive and well-established statistical system, this study utilizes annual data from 108 counties and districts in Guangdong Province, sourced from the *Guangdong Rural Statistical Yearbook* [60]. The data include grain production, population, sown area, yield, and per capita grain consumption for both urban and rural residents. The time range of the data spans from 2014 to 2023, according to the availability of information from the *Guangdong Rural Statistical Yearbook* [60].

## 3. Results

### 3.1. Structure and Trend of Grain Production

Clarifying the composition of grain is one of the critical challenges in grain-related research, especially in the context of its implications for food security and human behavior. In China, maize, wheat, rice, and potato are recognized as staple grains that serve as the cornerstone of national and regional food security. However, these grains differ significantly in terms of yield potential, farming systems, and socio-cultural influences. Such differences have inspired derivative studies on how grain production and consumption shape social behaviors. For instance, the “rice theory” suggests that the labor-intensive nature of rice farming—requiring almost twice as many the hours from seeding to harvest as wheat—has contributed to the development of collectivist behaviors in Southern China [61]. Conversely, the less labor-intensive wheat farming in Northern China is associated with more individualistic tendencies.

**Figure 3:**
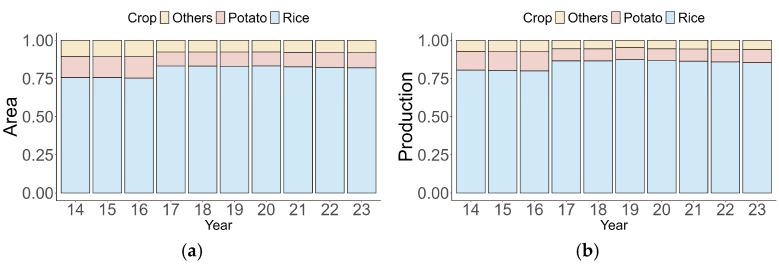
Structural composition and trends of grain-sowing area (**a**) and production (**b**) in Guangdong Province, 2014–2023. Data source: *Guangdong Rural Statistical Yearbook* [60].

Given these disparities, this study prioritized analyzing the structural composition of grain production in Guangdong Province to provide a precise understanding and avoid ambiguity. The figure illustrates the proportion of different grain crops (rice, potato, and others) in terms of sowing area and production from 2014 to 2023 (Figure 3). Throughout the period, rice consistently dominated, accounting for over 75% of the total grain production and sowing area each year. This highlights its critical role in maintaining food security in Guangdong Province. Meanwhile, potato and other grains occupied relatively minor shares, collectively accounting for less than 25%. Despite slight annual fluctuations, the structural composition of grain production and sowing area remained relatively stable over the study period, reflecting the limited diversification of crop production at the provincial level. Notably, the consistent dominance of rice highlights its strategic importance in regional agricultural planning and food policy, especially in urban areas where food security concerns are more pressing.

### 3.2. Self-Sufficiency of Grain

Table 1 presents the *GSSG* and *RSSG* for both grain-producing areas and the entire area of Guangdong Province under current yield conditions. The results reveal significant regional disparities in both *RSSG* and *GSSG*. In grain-producing areas, the RSSG ranges from 22.96% in the east to 45.76% in the north, with an average of 33.08% across all regions. In contrast, the *GSSG* shows a deficit across all regions, with the Pearl River Delta exhibiting the largest gap (−8.12 million tons), followed by the east (−5.85 million tons), west (−4.59 million tons), and north (−4.32 million tons). When considering the entire area of Guangdong, which includes non-agricultural districts that rely entirely on grain imports, the *RSSG* decreases slightly, with an average of 28.14%, falling short of the government’s target of 30%. The *GSSG* also worsens, particularly in the Pearl River Delta, where the deficit rises to −13.74 million tons.

**Figure 4:**
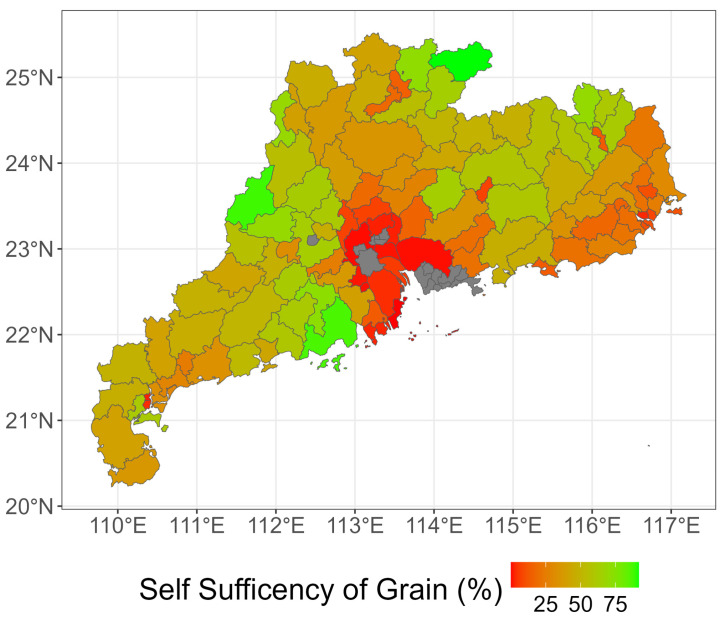
Spatial distribution of grain self-sufficiency in Guangdong Province. Data source: *Guangdong Rural Statistical Yearbook* [60].

The map (Figure 4) shows the spatial distribution of grain self-sufficiency (%) across Guangdong Province, highlighting significant regional disparities and distinct agglomeration patterns: The spatial distribution of grain self-sufficiency in Guangdong Province, as detailed in the figures, reveals significant regional disparities. The Delta region, which includes both urban districts and rural counties, shows a wide range of *RSSG*, indicating different levels of grain self-sufficiency. The east region also exhibits variations, with some counties showing high self-sufficiency and others indicating a need for external grain sources. The west region presents a mixed picture, with some districts and counties having high *RSSG*, indicating surplus grain production, while others have lower values, indicating a reliance on external supplies. The north region shows a spread in *RSSG*, with some counties standing out as highly self-sufficient.

The spatial analysis of grain self-sufficiency in Guangdong Province reveals a complex picture of regional disparities in grain production and consumption. The results indicate that while some districts and counties have a high capacity for grain self-sufficiency, others face significant challenges in meeting their grain needs. The yield gap between districts and counties, as indicated by the *RSSG*, is not statistically significant, suggesting that both urban and rural areas play a crucial role in the province’s food security. These insights highlight the importance of targeted strategies to enhance grain productivity, particularly in areas with lower *RSSG*, to ensure a more balanced and resilient food supply system across Guangdong Province.

### 3.3. Comparison of Grain Yield Between Guangdong and Other Provinces in China

**Figure 5:**
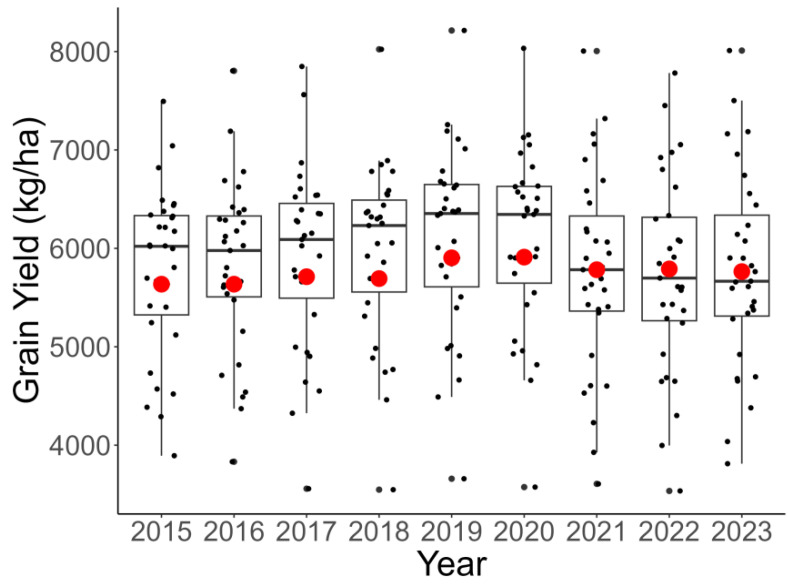
Comparison of grain yield between Guangdong Province and other provinces in China (2015–2023). The red dot in the figure highlights Guangdong’s grain yield. Data source: *China Statistical Yearbook* [40].

Figure 5 presents a comparative analysis of grain yield (measured in kg/ha) between Guangdong Province and other provinces in China from 2015 to 2023. The red dot in the figure highlights Guangdong’s grain yield, placing it within the broader context of China’s agricultural productivity. The figure reveals that Guangdong’s grain yield, while showing modest improvements over the years, remains below the upper quartile of grain-producing provinces in China. Over the nine-year period, Guangdong’s grain yield fluctuated between 5636 kg/ha in 2015 and 5911 kg/ha in 2020, with a slight decline to 5764 kg/ha in 2023. In contrast, provinces such as Jilin, Heilongjiang, and Jiangsu consistently achieved higher yields, with Jilin reaching 7494 kg/ha in 2015 and Heilongjiang achieving 6968 kg/ha in 2020. These provinces, which represent the upper quartile of grain-producing regions, demonstrate the potential for significantly higher agricultural productivity. The comparison highlights a notable gap between Guangdong’s grain yield and that of the leading provinces. For example, in 2023, Guangdong’s yield of 5764 kg/ha was substantially lower than those of Jilin (7186 kg/ha) and Liaoning (7164 kg/ha), despite the latter experiencing a decline in recent years. The figure also emphasizes the importance of closing this yield gap to ensure food security in Guangdong Province, one of China’s most economically developed and densely populated regions; it also illustrates the potential for Guangdong to improve its grain yield and align with the leading grain-producing provinces in China.

### 3.4. Enhancing Grain Self-Sufficiency: Scenarios and Projections in Guangdong Province

Most existing studies focus on identifying the risk of regional grain self-efficiency, and this study makes further exploration by simulating the change in self-sufficiency of grain under different scenarios. Table 2 presents the projected changes in the rate of grain self-sufficiency (RSSG) for Guangdong Province under four distinct scenarios: Scenario A (baseline), Scenario B (grain loss reduction), Scenario C (grain yield improvement), and Scenario D (integrated scenario combining grain loss reduction and yield improvement). The simulations are conducted at both regional and provincial levels, covering the Pearl River Delta, east, north, and west Guangdong.

Scenario A (baseline): This scenario reflects the current conditions of grain production and loss, with an *RSSG* of 28.14% for the entire province. Regional disparities are evident, with the north exhibiting the highest *RSSG* (45.55%) and the Pearl River Delta having the lowest (16.58%). These results align with the findings presented in Table 1, serving as a reference point for assessing the potential impacts of the proposed interventions.

Scenario B (grain loss reduction): In this scenario, grain loss in various regions of Guangdong Province decreases from the national average of 12%, reflecting Guangdong’s potential to reduce post-harvest losses. The grain loss rate for different regions is set based on factors such as infrastructure, storage conditions, and transportation networks. The northern region, characterized by generally high elevations and mostly mountainous terrain, faces higher loss rates. In contrast, the southern region has flat terrain, dominated by plains and terraces. The Pearl River Delta is the largest plain in the province, followed by the Chaoshan Plain in the east [62]. Due to well-developed logistics facilities and advanced storage technologies, grain loss is lower in the eastern and Pearl River Delta regions. However, the loss rate is higher in the western and northern regions due to poor terrain conditions, inconvenient transportation, and relatively weak infrastructure [63,64]. Based on this, the Pearl River Delta region is assigned the lowest loss rate (3%), followed by the eastern region (4%), and then the western and northern regions, which have higher loss rates (5%, 5%).

The simulation results show a moderate increase in *RSSG* across all regions, with the provincial average rising to 30.58%. The north experiences the most significant improvement, with its *RSSG* increasing to 49.17%, while the Pearl River Delta experiences a more modest rise to 18.27%. This scenario highlights the importance of reducing grain loss as a viable strategy to enhance grain self-sufficiency.

Scenario C (grain yield improvement): This scenario assumes that Guangdong’s grain yield can be increased. Different grain yield coefficients are set for each region based on factors such as terrain conditions, arable land resources, and agricultural technology levels. For example, in terms of arable land pressure, northern Guangdong has the highest pressure, followed by the Pearl River Delta and eastern Guangdong, with the lowest pressure in western Guangdong [36]. Additionally, the overall spatial distribution of arable land loss at the county level in Guangdong shows a trend of gradual increase from south to north and from west to east. The northern region has less arable land due to a higher proportion of mountainous and hilly areas. The Pearl River Delta plain is continuously losing arable land due to the influence of the economic zone, resulting in insufficient reserve land. In contrast, the coastal areas of eastern Guangdong, such as the Chaoshan Plain and the Leizhou Peninsula–Dianbai–Yangjiang area in the western coastal region of Guangdong Province, have relatively larger amounts of arable land [65,66]. Based on these differences, we have set different grain yield coefficients for each region. Eastern Guangdong has the highest yield (12%), aligning with the upper quartile of grain-producing provinces in China (as shown in Figure 4), followed by the Pearl River Delta (10%), and then western and northern Guangdong with lower yields (8%, 8%).

The results indicate a further improvement in *RSSG*, with the provincial average reaching 30.7%. The north again benefits the most, achieving an *RSSG* of 49.19%, while the Pearl River Delta improves to 18.23%. This scenario highlights the potential for yield improvement through technological progress and improved agricultural practices.

Scenario D (integrated scenario): Combining the effects of grain loss reduction and yield improvement, Scenario D represents the most optimistic projection. The provincial *RSSG* rises to 34.14%, exceeding the government’s target of 30%. Regionally, the north achieves the highest *RSSG* at 53.1%, followed by the west (46.52%), the east (27.58%), and the Pearl River Delta (20.1%). This integrated approach demonstrates the synergistic effects of simultaneously addressing grain loss and yield improvement, providing a comprehensive pathway to enhance grain self-sufficiency in Guangdong.

Hence, Table 2 illustrates the potential for Guangdong to significantly improve its grain self-sufficiency through targeted interventions. The results suggest that a combination of grain loss reduction and yield improvement can elevate the provincial *RSSG* to 34.14%, thereby contributing to food security and aligning with the national “dual circulation” strategy. These results provide a clear roadmap for policymakers and stakeholders to prioritize investments in agricultural technology and infrastructure, ensuring a stable and resilient grain supply for Guangdong’s growing population.

## 4. Discussion and Policy Recommendation

### 4.1. Storing Grain in the Land: Farmland Reservation for Grain Supply

China has experienced rapid and massive urban expansion in recent decades, and several studies have assessed the cropland loss due to settlement. Wang et al. (2019) [67] estimated that 5.92 million hectares (ha) or 3.31% of cropland were lost from 2000 to 2010. Huang et al. (2019) [68] found that the annual expansion and annual growth rate of farmland occupation were nearly 1900 ha and 5% in 291 Chinese cities during 1990–2015. Wu et al. (2024) [69] calculated that, from 1990 to 2020, approximately 4000 hectares (ha) of farmland were occupied annually. In order to mitigate the continuous farmland occupation from urbanization and industrialization, Chinese government stipulates that the limit of 1.8 billion mu (120 million ha) of farmland should be maintained to ensure national food security [57,70,71], which is called “Red Line of Farmland Protection Policy”. In 2015, this policy has been emphasized by the “storing grain in the land” strategy [72]. Apart from urban expansion, a significant proportion of farmland has been converted for ecological restoration, through initiatives such as the grain-for-green programs [73,74,75]. Auch approaches can protect environmental and ecological systems, thereby ultimately enhancing the resilience of agricultural production. The trade-off between worries of short-term grain [73,75] and long-term agricultural sustainability suggests that these programs could bring substantial social and ecological benefits [74].

According to the trend in the sown area of grain in Guangdong, it has remained stable for a long time between district and county. This is because the Guangdong government has demonstrated a heightened commitment to farmland preservation by implementing stringent policies, which emphasize the maintenance of the red line of farmland protection, thereby reinforcing the foundation for sustainable food security [76].

### 4.2. Storing Grain in Technology: Improvement of Grain Yield

The “storing grain in the technology” strategy is a critical component in China’s efforts to enhance agricultural productivity and ensure food security [77,78,79]. While the “storing grain in the land” policy focuses on preserving arable land, the technological approach emphasizes innovation to boost grain yields. However, as the comparative analysis of grain yields between Guangdong Province and other provinces reveals, a significant yield gap persists, highlighting both the challenges and opportunities for Guangdong Province to achieve higher agricultural productivity.

Although Guangdong’s grain yield has shown some improvements in recent years, it remains below the upper quartile of grain-producing provinces such as Jilin and Heilongjiang. This gap highlights the urgent need to improve grain yield, which requires the adoption of advanced agricultural technologies [80], improved resource management [81], and stronger policy support [82]. Specific strategies include investing in precision farming technologies such as remote sensing, data analysis, and automated machinery to optimize resource use and increase yields. Additionally, improving soil quality through sustainable practices, optimizing irrigation systems, and promoting crop diversification can contribute to more stable and higher grain yields. Strengthening support for farmers through policy measures, financial incentives, and education on best agricultural practices is also essential. By improving its grain yield, Guangdong could enhance its food supply, contribute to the national “dual circulation” strategy, and strengthen its resilience against food supply disruptions, ultimately achieving food self-sufficiency.

The recent “New Round of Action Plan for Increasing Grain Production Capacity by 100 Billion Jin (2024–2030)”, issued by the State Council in 2023, provides a timely framework for addressing these challenges [83]. The plan sets an ambitious target of increasing grain production capacity by 50 million tons by 2030, emphasizing the need for breakthroughs in several key areas: efficient utilization of arable land, optimization of planting structures, prevention of natural disasters, and reduction in grain processing loss. These priorities are closely aligned with Guangdong’s current agricultural challenges, particularly in the context of its rapid urbanization and industrialization, which have placed significant strain on its limited agricultural resources.

One of the most promising aspects of the “grain storage through technology” strategy is the development and promotion of high-yield and high-quality crop varieties. In recent years, China has made significant progress in this area; Guangdong Province has also strengthened farmland protection and land use management, implementing multiple measures to enhance farmers’ enthusiasm for grain production. The promotion of high-yield and high-quality varieties, the implementation of site-specific practices such as reasonable planting density, and the application of technologies like “one spray, multiple promotions” have all contributed to increasing grain yields and continuously enhancing food security [84]. However, compared to other provinces, Guangdong Province still faces some challenges in the area of food security technology. The growing constraints of resources and the environment, such as soil degradation and water scarcity, are particularly severe in Guangdong due to its high population density and intensive land use [85]. Furthermore, climate change has intensified resource–environmental pressures in the region, while the frequent occurrence of natural disasters poses a significant threat to grain production. To address these challenges, China’s Grain Production Capacity Enhancement Action Plan proposes several reasonable solutions, including the improvement of soil quality, advancement in the construction of high-quality farmland, and optimization of planting patterns, with an emphasis on concentrating high-yield crops in favorable production areas [86]. These strategies can not only increase productivity, but also integrate environmental and ecological protection to achieve dual goals of food security and sustainable development. Specifically, by combining technological innovations with ecological measures—such as promoting water-saving irrigation technologies and constructing high-quality farmland—it is possible to increase grain yields while restoring and improving soil quality, alleviating water shortages, and enhancing the resilience of agricultural systems to climate change and natural disasters. This integrated approach, which prioritizes both “grain storage through technology” and ecological conservation, provides a sustainable pathway toward food sovereignty, ensuring the stability and resilience of grain production in Guangdong Province despite the pressures of climate change and resource–environmental constraints.

For Guangdong Province, these strategies provide a clear pathway for improving grain yield and achieving self-sufficiency. However, strategies must also address the broader socioeconomic factors that have hindered agricultural innovation. For instance, the rapid urbanization and industrialization of Guangdong have led to a shift in labor and investment away from agriculture, creating a need for policies that incentivize technological adoption and promote sustainable farming practices. Moreover, the province’s reliance on grain imports from other regions highlights the urgency of enhancing local production capacity to reduce vulnerability to external supply disruptions.

In conclusion, while the “storing grain in the technology” strategy provides a robust framework for improving grain yields, its success in Guangdong Province will depend on the province’s ability to address both technological and structural challenges. By leveraging national policies and investing in agricultural innovation, Guangdong Province can close the yield gap with leading grain-producing provinces, enhance its food security, and contribute to the broader national agenda of sustainable agricultural development. This dual focus on technology and policy will be essential for ensuring stable and resilient food supply in the face of growing internal and external pressures.

### 4.3. Grain Loss Reduction

Grain loss occurs during the stages of sowing, cultivation, harvesting, post-harvest handling, storage, and distribution [87]. Grain loss reduction is a critical component of enhancing food security, particularly in a high-density and economically developed region like Guangdong Province. The province faces significant challenges in minimizing post-harvest losses. Reducing these losses is not only essential for improving grain self-sufficiency but also aligns with the national “dual circulation” strategy, which emphasizes domestic production and resilience against external disruptions.

One of the major challenges in reducing grain loss is the impact of natural disasters. China loses millions of tons of grain annually due to extreme weather events such as droughts, floods, and typhoons [88]. In Guangdong Province, these events occur frequently due to its unique geography and climate, making it crucial for local governments to refine flood prevention and preparedness measures to enhance overall disaster response capabilities [89]. Recently, several regions in Guangdong Province have proactively planned for the 2025 flood prevention work, including hazard investigation and remediation, improving emergency disaster relief supply systems, and bolstering emergency material support during extreme events [90]. Although Guangdong Province has made significant progress in agricultural disaster prevention, it is recommended that the province strengthen meteorological monitoring and early warning systems to improve the accuracy and timeliness of disaster alerts [91]. In addition to preventive measures, effective crisis management strategies need to be implemented to minimize the impact of disasters on grain production. This includes flood control infrastructure such as reservoirs and barriers and ensuring that irrigation systems are capable of managing both drought and excessive rainfall. Furthermore, disaster-resistant agricultural technologies, such as drought-tolerant crop varieties and improved drainage systems, should also be promoted. Rapid post-crisis recovery plans should be in place, providing financial support, agricultural subsidies, and technical assistance for replanting and soil recovery. By strengthening both disaster prevention and recovery strategies, Guangdong can reduce the vulnerability of its grain production to climate-related disruptions and ensure a more resilient food supply [92].

In addition to natural disaster prevention, it is equally important to focus on reducing loss during grain processing, storage, and transportation. These stages of the supply chain are often overlooked but can result in substantial grain loss. For example, inefficient storage facilities, inadequate infrastructure, and poor transportation networks can lead to spoilage, contamination, and waste [93]. To address these challenges, the People’s Government of Guangdong Province issued the 2022 Implementation Plan for Grain Saving and Loss Reduction. The plan focuses on strengthening grain saving and loss reduction across various stages of the supply chain, including agricultural production, grain storage, transportation, and processing. It also emphasizes enhancing public education and awareness on grain saving and loss reduction [94]. At present, measures for grain saving and loss reduction should be further strengthened and detailed across all stages of the supply chain. Technological innovation plays a key role in minimizing grain loss. The development of high-efficiency drying and storage technologies can help preserve grain quality and reduce post-harvest losses. Additionally, integrating digital tools and data analytics into the supply chain can improve monitoring and decision-making processes. Real-time data tracking can detect problems early and adjust operations accordingly, thus reducing losses [95].

Strategic policy interventions, such as financial support for infrastructure upgrades, training for better management practices, and the development of digital supply chain platforms, are critical in enhancing the effectiveness of these strategies [96]. By combining technological advancements with targeted policies, Guangdong Province can significantly reduce grain loss and improve overall food security. This integrated approach will not only improve the efficiency of grain storage and transportation, but also contribute to more sustainable food systems.

### 4.4. Interdepartmental Coordination

The successful implementation of food production and technological innovation policies, as discussed in Section 4.1, Section 4.2 and Section 4.3, depends on Guangdong’s ability to address broader socioeconomic and infrastructure challenges contributing to grain loss. Additionally, Guangdong Province needs to focus on public policy areas, particularly urban planning and land protection. Rapid urbanization and industrialization have led to the occupation of large areas of farmland, reducing the amount of land available for grain production. A balance between urbanization and land protection is needed to ensure food security [97].

As China’s largest province in terms of economy and population, Guangdong Province has made the strict protection of arable land a key political objective. The “Special Plan for Arable Land Protection in Guangdong Province (2021–2035)” was developed to guide the remediation of arable land, improve land quality, and support high-quality development in counties, towns, and villages [98]. It is also important to ensure the long-term stability of land for food production, prevent illegal or excessive land development, and promote efficient land use to avoid unregulated development that could affect grain production [99].

Furthermore, it is essential to enhance land reclamation and transfer policies. Some agricultural land may be left unused during urbanization, and an appropriate land transfer mechanism can improve land use [100]. The government should also encourage farmers to transfer land to support modern agricultural development and enhance land productivity.

Infrastructure development is also crucial for ensuring food supply. With urbanization, the construction of logistics and storage facilities becomes increasingly important [101]. Guangdong Province has held meetings to strengthen coordination on food security [102]. However, inter-departmental cooperation remains a challenge. The government needs to integrate policies on agriculture, urban planning, environmental protection, and economic development for unified action. This collaboration will promote land protection, sustainable agriculture, and food security. By raising public awareness of land resource protection, society can foster harmonious urban–rural development and ensure the sustainability of food production. As shown in Table 3, based on the analysis results of this study, the policy recommendations for the above aspects are summarized.

## 5. Conclusions

This study has comprehensively examined the grain self-sufficiency in Guangdong Province, focusing on the spatial and temporal patterns of the *RSSG* from 2014 to 2022. The findings reveal that Guangdong Province faces significant challenges in achieving its grain self-sufficiency target, with an average *RSSG* of 28.14%, falling short of the government’s goal of 30%. The study also highlights significant regional disparities, with the Pearl River Delta region exhibiting the lowest *RSSG* due to its high population density and limited agricultural land. In contrast, the north region demonstrates a higher RSSG, primarily due to its relatively lower population density and greater agricultural capacity.

The analysis of grain production structure indicates that rice remains the predominant grain crop in Guangdong Province, accounting for over 75% of the total sowing area and production. This highlights the critical role of rice in maintaining food security in the province. However, the yield gap between Guangdong Province and other leading grain-producing provinces in China suggests that there is significant potential for improvement through technological advancements and policy support.

Scenario simulations show that improving grain yield and reducing grain loss could significantly increase the grain self-sufficiency rate, potentially raising it to 34.14%. This finding highlights the importance of implementing a dual strategy of “storing grain in the land” and “storing grain in the technology” to address challenges in farmland preservation and agricultural productivity. Specifically, this includes strengthening farmland protection policies, promoting sustainable agricultural practices, and investing in advanced agricultural technologies. Additionally, the study proposes several other policy recommendations, such as improving post-harvest management, enhancing storage facilities, and strengthening disaster prevention measures to reduce grain loss. It also emphasizes the need for strengthening cross-sectoral coordination, fostering collaboration between agriculture, environmental, and urban planning departments, and collectively promoting food security and higher self-sufficiency in grain production.

However, in implementing these measures, we may face some challenges. For example, in strengthening farmland protection, finding approaches for balancing urbanization with farmland preservation, encouraging farmers to adopt high-yield technologies, and improving infrastructure with limited resources are all difficulties that need to be overcome. Additionally, Guangdong’s grain self-sufficiency rate is influenced by factors such as natural disasters, climate change, and fluctuations in the global supply chain, all of which require the government to adopt flexible responses.

The research methods and results are not only applicable to Guangdong Province but also provide valuable insights for other countries or regions, particularly those facing similar urbanization processes, agricultural production issues, and food security challenges. Future research could further explore the applicability of grain self-sufficiency policies in other countries, especially how to adjust policies based on different countries’ economic conditions, social structures, and climatic characteristics. This would help provide more practical experiences and references for global food security and sustainable agricultural development.

In the context of increasing global uncertainties, such as natural disasters, epidemics, and international conflicts, the findings of this study highlight the urgency of enhancing Guangdong’s grain self-sufficiency. By improving the level of grain self-sufficiency, Guangdong can contribute to China’s overall food security strategy and reduce its vulnerability to external supply disruptions. The insights provided in this study offer a clear roadmap for policymakers and stakeholders, guiding them to prioritize investments in agricultural technology and infrastructure to ensure the stable and resilient food supply for Guangdong’s growing population. Future research should focus on exploring the socioeconomic and environmental impacts of grain self-sufficiency policies and finding innovative solutions to address the challenges of sustainable agricultural development in the context of rapid urbanization and climate change.

## Figures and Tables

**Table 1:** Gap and rate of grain self-sufficiency (*GSSG*, *RSSG*) in grain-producing areas and the entire area of Guangdong under current yield.

Region	Grain-Producing Area		Whole Area	
	*GSSG*	*RSSG*	*GSSG*	*RSSG*
Delta	−8.12	25.17	−13.74	16.58
East	−5.85	22.96	−5.98	22.57
North	−4.32	45.76	−4.36	45.55
West	−4.59	41.01	−4.8	39.9
All	−22.88	33.08	−28.89	28.14

Units: *GSSG* (million tons); *RSSG* (%).

**Table 2:** Simulation of change of rate of grain self-sufficiency (*RSSG*) under different scenarios.

Region	Scenario A	Scenario B	Scenario C	Scenario D
Delta	16.58	18.27	18.23	20.1
East	22.57	24.63	25.28	27.58
North	45.55	49.17	49.19	53.1
West	39.9	43.07	43.09	46.52
All	28.14	30.58	30.7	34.14

**Table 3:** Summary of policy recommendations for promoting grain self-sufficiency in Guangdong Province.

Policy Name	Policy Goal	Policy Measures
Storing Grain in the Land: Farmland Reservation for Grain Supply	Protect farmland resources and ensure food supply.	Strengthen farmland protection policies. Establish the “Red Line of Farmland Protection”. Limit the conversion of arable land.
Storing Grain in Technology: Improvement of Grain Yield	Increase grain yield and enhance agricultural productivity.	Improve resource management. Adopt advanced agricultural technologies. Strengthen policy support and enhance farmer training.
Grain Loss Reduction	Reduce grain loss from production to consumption.	Prevent natural disasters and ensuring post-disaster recovery. Improve storage and transportation networks within the supply chain. Develop and implement efficient drying and storage technologies.
Interdepartmental Coordination	Strengthen coordination between government departments and promote policy implementation.	Optimize urban planning policies. Strengthen the implementation of land reclamation and land transfer policies. Enhance infrastructure construction and raise public awareness.

## Data Availability

The raw data supporting the conclusions of this article will be made available by the authors on request due to specific reason or restrictions.

## Abbreviations

The following abbreviations are used in this manuscript:

RSSG	rate of grain self-sufficiency
GSSG	gap of grain self-sufficiency
